# Assessing placental membrane treatment efficiency in diabetic foot ulcers: Processing for retention versus lamination

**DOI:** 10.1002/hsr2.2196

**Published:** 2024-06-17

**Authors:** Robert G. Frykberg, Zwelithini Tunyiswa

**Affiliations:** ^1^ Open Wound Research Puyallup Washington USA

**Keywords:** amniotic tissues, diabetic foot ulcers, wound healing

## Abstract

**Background:**

Diabetic foot ulcers are a severe complication in diabetic patients, significantly impact healthcare systems and patient quality of life, often leading to hospitalization and amputation. Traditional Standard of Care (SOC) treatments are inadequate for many patients, necessitating advanced wound care products (AWCPs) like human placental membranes. This study conducts a retrospective analysis to compare the effectiveness of two human placental membrane products, retention‐processed amnion chorion (RE‐AC) and lamination‐processed amnion chorion (L‐AC) in managing chronic diabetic foot ulcers (DFUs).

**Methods:**

The study collected retrospective observational data from electronic health records (EHRs) of patients treated for DFU at three outpatient wound care centers. Patients were categorized into two cohorts based on the treatment received. Key metrics included wound size progression and the number of product applications. The analysis employed Bayesian estimation, utilizing an analysis of covariance model with a Hurdle Gamma likelihood.

**Results:**

We found that RE‐AC achieved a marginally higher expected Percent Area Reduction (xPAR) in DFUs compared to L‐AC at 12 weeks (67.3% vs. 52.6%). RE‐AC also required fewer applications, suggesting greater efficiency in general wound closure. Probability of full wound closure was similar in both groups (0.738 vs 0.740 in RE‐AC and L‐AC, respectively).

**Conclusion:**

The findings suggest that while L‐AC might be slightly more effective in complete ulcer healing, RE‐AC offers overall better treatment efficiency, especially in reducing the frequency of applications. This efficiency can lead to improved patient comfort, reduced treatment costs, and optimized resource utilization in healthcare settings.

## 1 INTRODUCTION

The complications of diabetes mellitus (DM) and concurrent health conditions have adverse effects on the process of wound healing. This contributes to the formation of chronic diabetic foot ulcers (DFUs), which are characterized by ulcers that do not follow the usual healing stages within an expected timeframe and remain open for an extended duration. DFUs are one of the most prevalent and severe complications affecting the lower extremities in individuals with DM. They pose a significant economic strain on healthcare systems, with annual treatment costs estimated to range from $9 to $13 billion (about $40 per person) in the United States alone.^1^

These chronic DFUs have detrimental consequences, including reduced quality of life for patients, heightened risks of infection, amputation, and even mortality. The duration of DFUs represents an independent risk factor for foot infections, which in turn are linked to a substantially elevated risk of hospitalization (55 times greater) and amputation (155 times greater.^2^ DFUs are important components in the pathway to amputation and precede approximately 85% of diabetes related lower extremity amputations.^2^ Furthermore, ulcerations and amputations resulting from diabetes have 5‐year mortality rates that are on par with colon cancer and exceed those of prostate and breast cancer.^3^

Accumulated data consistently demonstrates that DFUs have profoundly adverse effects on patients' quality of life and place substantial burdens on social, economic, and healthcare systems.^4^

Managing DFUs continues to present a formidable challenge for healthcare providers. Effective wound care commences with a comprehensive evaluation of both the patient and the wound itself, followed by the implementation of Standard of Care (SOC) measures. This includes essential steps such as debridement, infection control, the application of moist dressings, and the alleviation of pressure on high‐stress areas.

Relying solely on SOC, nearly 70% of DFUs do not achieve healing even after 20 weeks of treatment.^5^ Diabetic patients, particularly those of advanced age with underlying conditions like peripheral neuropathy, ischemia, and peripheral arterial disease (PAD), face a heightened risk of SOC treatment failure. These high‐risk individuals encounter difficulties in the wound healing process and often require advanced interventions that have the potential to facilitate wound closure.

Cellular and acellular matrices, also known as skin substitutes, form a wide‐ranging category of advanced wound care products (AWCP) designed to facilitate the natural wound healing process. These products are typically employed as supplementary treatments when a patient does not respond adequately to SOC alone. Examples of AWCPs encompass bioengineered living cellular and dermal constructs, skin allografts, and a diverse array of collagen matrices derived from animals. A recent expert consensus panel introduced a new term for these cellular, acellular, and matrix‐like products (CAMPs).^6^ Over the last decade, human placental membranes have been widely incorporated into wound care protocols in the United States and comprise a significant proportion of those CAMPs used for managing chronic nonhealing DFUs.

Placental membranes possess properties that support the natural wound healing process, including anti‐inflammatory, antimicrobial, anti‐fibrotic, and angiogenic characteristics.^7^, ^8^ A recent review of wound healing with placental membranes^9^ highlights the processing effects of cryopreservation and dehydration on potential factors available to wound beds.

John Davis was the first to report (1910) the use of amnion membrane as a surgical material in skin transplantation. He showed that in skin grafting, amniotic membrane (AM) resulted in better outcomes than xenograft or cadaveric dressings.^10^ Since then, human amniotic membrane has been used widely in regenerative medicine due to continued discovery of its favorable biological and mechanical properties.^11^, ^12^, ^13^

When amnion and chorion are used together, the chorion, which is thicker, is reported to be responsible for 75% of the growth factors present.^11^ Amnion with chorion (AC) in various forms have been used for numerous applications. The primary application to date is for wound healing.^14^, ^15^, ^16^ For example, in 2015 Zelen et al.^17^ reported that use of laminated dehydrated human AC resulted in better and faster healing in wound care compared to other products. Other reports include AC in dental and oral treatments,^18^, ^19^, ^20^ urogenital system issues,^21^, ^22^, ^23^ and orthopedic treatments.^24^, ^25^ As seen with AM, there is documented antimicrobial activity from AC.^26^, ^27^ It is well documented that placental membranes, including AC contain immunomodulatory factors.^28^ These include IL‐1ra,^29^, ^30^ HGF,^31^, ^32^ and PDGF‐BB.^33^, ^34^ The processing of placental membranes, and subsequent retention of these factors, depends on the desired application. Membranes that are exposed to a series of chemicals to remove cells, proteins, lipids, etc., render a membrane that is structurally sound for fibroblast infiltration during the healing process. Other processing methods separate the membranes (amnion from chorion), process each individually, re‐laminate them back together, dehydrate and sterilize (usually gamma irradiate). In this manuscript we refer to this process as L‐AC. This produces a structurally sound, sterile ablated product with excellent handling characteristics. A different method of processing involves minimal processing of the membrane, cold neutral washing buffers, gentle dehydration and gentle e‐beam sterilization. In this manuscript we refer to this process as RE‐AC. This produces a structurally sound, sterile, growth factor‐rich product with equally amenable handling characteristics. While we have not measured residual DNA in the products, it is well accepted that storing tissue frozen before processing (freeze/thaw) and e‐beam sterilization post processing will fragment, degrade and crosslink DNA. There is the possibility that DNA fragments, perhaps crosslinked, exist in the membranes. Nonetheless, all processing of human placental tissues stringently follow industry and government standards. The risk of transmission of infectious (or other) agents is minimized through careful donor screening of all donated placental tissue, aseptic manufacturing technologies, adhering to current Good Tissue Practice and current Good Manufacturing Practice requirements, as well as terminal sterilization.

A retrospective analysis evaluating the effectiveness of a sterile dehydrated amnion/chorion membrane processed using the BioREtain® method (RE‐AC) versus a sterile dehydrated amnion/chorion method processed by a more ablative method (L‐AC) was performed using retrospective observational data collected from electronic health record (EHR) data from three^3^ outpatient wound care centers. Since this was a deidentified retrospective review of previously collected data, IRB waiver was granted.

## 2 MATERIALS AND METHODS

### 2.1 Study design and data collection

In this retrospective analysis, the primary objective was to evaluate and compare the effectiveness of two wound care products for managing a variety of chronic wounds: RE‐AC (AmnioWrap2®) processed using the BioREtain® method (BioStem Technologies) and L‐AC (Epifix®) processed by a more ablative method (Mimedix). The analysis was based on the utilization of retrospective observational data obtained from EHRs from three different outpatient wound care centers.

Initially, the research team identified a pool of potential study participants who met specific inclusion criteria. A total of 41 subjects were identified who met the study's inclusion criteria. These criteria included factors such as the type of wound (DFU), medical history, and treatment history that were relevant to the research question. This step was essential to ensure that the selected subjects were appropriate for the study (Table 1).

**Table 1:** Demographic overview of cohorts.

		RE‐AC	L‐AC
Subjects		23	18
Age		71.0	58.6
		(11.7%)	(8.1%)
Sex	Female	5 (22%)	3 (17%)
	Male	18 (78%)	15 (83%)
Race	Asian	2 (9%)	0 (0%)
	Black	1 (4%)	1 (6%)
	Hispanic	8 (35%)	7 (39%)
	Native Hawaiian or Other Pacific Islander	1 (4%)	0 (0%)
	White	3 (13%)	2 (11%)
	Unknown	8 (35%)	8 (44%)

Abbreviations: L‐AC, amination‐processed amnion chorion; RE‐AC, retention‐processed amnion chorion.

The researchers gained access to the EHR of 41 identified subjects from three outpatient wound care centers. EHRs contain comprehensive medical information about patients, including their medical history, diagnoses, treatments, and clinical notes. This data source allowed for a detailed retrospective analysis of the subjects' medical journeys.

Once the relevant patient records were accessed, the 41 subjects were categorized into two cohorts: the RE‐AC cohort (comprising 23 subjects) and the L‐AC cohort (comprising 18 subjects) as seen in the demographical overview Table 1. This categorization was based on the specific treatment each patient had received during their wound care. The study team extracted various data points from the EHRs for all 41 subjects. The most critical data included wound size in square centimeters at multiple time points. This information provided a quantitative measure of the wound's progression or healing over time. Additionally, the frequency of applications for each product was documented within their respective cohorts.

Data was collected at sequential time intervals for all 41 subjects to track the changes in wound size, outcomes, and treatment applications as shown in Table 2. Quality control measures were implemented to ensure the data extracted from the EHRs were reliable and consistent. This involved cross‐referencing data, resolving discrepancies, and addressing missing information.

**Table 2:** Wound and treatment summary.

		RE‐AC	L‐AC
Starting area (in cm^2^)	Mean	16.5	14.2
(*Gamma Likelihood*)		(10.9–26.1)	(8.4–24.9)
	Variance	0.8	0.8
		(0.5–1.3)	(0.4–1.3)
Product applications	Mean	7.9	10.6
(*Negative Binomial Likelihood*)		(6.5–9.6)	(8.6–12.9)
	Variance	8.3	10.9
		(3.3–23.0)	(3.8–35)
Treatment days	Mean	68.2	77.3
(*Negative Binomial Likelihood*)		(55.7–84.9)	(66.8–89.1)
	Variance	3.8	12.5
		(2.0–6.7)	(4.9–28.5)
Probability of full wound closure	Mean	0.738	0.740
(*Hurdle‐Gamma Likelihood*)		(0.565–0.892)	(0.572–0.896)
Expected percent area reduction	Mean	67.3%	52.6%
(xPAR)		(49.2%–83.0%)	(15.6%–80.9%)
(*Hurdle‐Gamma Likelihood*)			

*Note*: Bayesian analysis was performed—estimates and 95% credible intervals (not confidence intervals) are reported

Abbreviations: L‐AC, amination‐processed amnion chorion; RE‐AC, retention‐processed amnion chorion.

### 2.2 Amnion with chorion description

Donated human placentas were acquired from accredited Gift of Life tissue recovery agencies after planned cesarean sections with informed consent. All donations and processing were completed in accordance with FDA Good Tissue Practices (GTP) and American Association of Tissue Banks (AATB) standards. Donors were screened for medical and social issues, communicable diseases, as well as infectious diseases, including human immunodeficiency virus (HIV), human T‐lymphotropic virus (HTLV), hepatitis B and C, syphilis, and cytomegalovirus (CMV). Additionally, grafts are terminally sterilized by electron beam sterilization.

Amnion with chorion was isolated from the placenta and processed with a proprietary BioRetain® procedure. This method processes the membrane intact, retaining the intermediate layer and uses balanced solutions for washing. The resulting dehydrated amnion/chorion (RE‐AC; Biostem Technologies) is cut to preferred sizes, packaged, and sterilized by e‐beam irradiation.

The comparator dehydrated amnion/chorion matrix product was similarly obtained but was processed with a different proprietary method. This method includes separation of the amnion and chorion, cleaning of the layers individually, and re‐lamination of the amnion and chorion before dehydration. The membrane is then sterilized by gammas irradiation. Both sterile products were prepackaged and available at different sizes without needing special refrigeration or freezer storage.

### 2.3 Statistical analysis

The researchers employed a Bayesian regression analysis in using PYMC (Probabilistic Programming in Python) estimating the treatment efficacy of RE‐AC relative to L‐AC in wound care management.^35^

Given the data‐generating process of the dependent variable (viz., wound area), which leads to either right‐skewed continuous data, or zero (i.e., a closed wound), the researchers utilized a Hurdle Gamma analysis of variance (ANCOVA) model to estimate expected percent area reduction (xPAR) from baseline. The model utilizes a baseline covariate adjustment to control for starting area between wounds. ANCOVA with baseline covariate adjustment has been shown to have the highest statistical power for analyses that are focused on change from baseline.^36^ The model provides the joint probability of both a wound being closed and the expected area of a wound at the posttimepoint. Under the Bayesian paradigm, we not only estimate the full posterior estimates of the likelihood, but we also get full uncertainty estimates in the parameters of the likelihood (the Hurdle Gamma Model). These parameters for the logistic binomial are Ψ (Psi), which is the probability of the wound measurement being zero (or closed), and the parameters for the Gamma component (surface area) are *α* (alpha—the dispersion parameter) and *β* (beta—the mean parameter). In small sample, and quasi‐experimental studies, like this one, the ability to generate full distributional estimates of parameters in addition to likelihood, allows for more robust inference, and flexibility in computing marginal effects.

The Hurdle Gamma model uses an ANCOVA setup because this allows for the control of baseline (pre) measurements, or covariate adjustment when estimating the closure rate and expected Percentage Area Reduction (“xPAR”) between groups. This is not possible in a Difference in Differences approach, which uses percentage change directly in the model. ANCOVA increases statistical power and precision by accounting for within‐group variability and removes confounder bias by adjusting for pre‐existing differences between groups.

This is a more robust approach that returns posterior estimates for the probability of a closed wound and expected Percent Area Reduction (xPAR) for both the RE‐AC and L‐AC groups. By adopting this rigorous approach, we can systematically examine and compare the treatment outcomes between the two products, ultimately yielding scientifically grounded insights into their respective efficiency. This analytical approach not only enables a robust comparison of treatments but also offers the opportunity to control potential confounding variables, thereby enhancing the precision of our findings.

The mathematical notation for the model is as follows

The Hurdle Component (the probability of a closed wound)

$${I(y}_{i}>0)\sim \;{\text{Bernoulli}(\pi }_{i}),$$

(1) $$\text{logit}(\pi_{i})=\alpha +\beta_{\times }\text{group}+\gamma \times {\text{pre}}_{i}.$$

The Gamma Component (Expected Percent Area Reduction (xPAR)),

(2) $$(y_{i}-pre_{i})|\;I(y_{i}>0)∼\;\text{Gamma}(\text{shape}=k,\text{rate}=k\;/\;{µ}_{i})\text{log}({µ}_{i})=\;\theta +\delta \text{group}\times {\text{group}}_{i}\;+\varepsilon \times {\text{pre}}_{i}.$$

**Figure 1:**
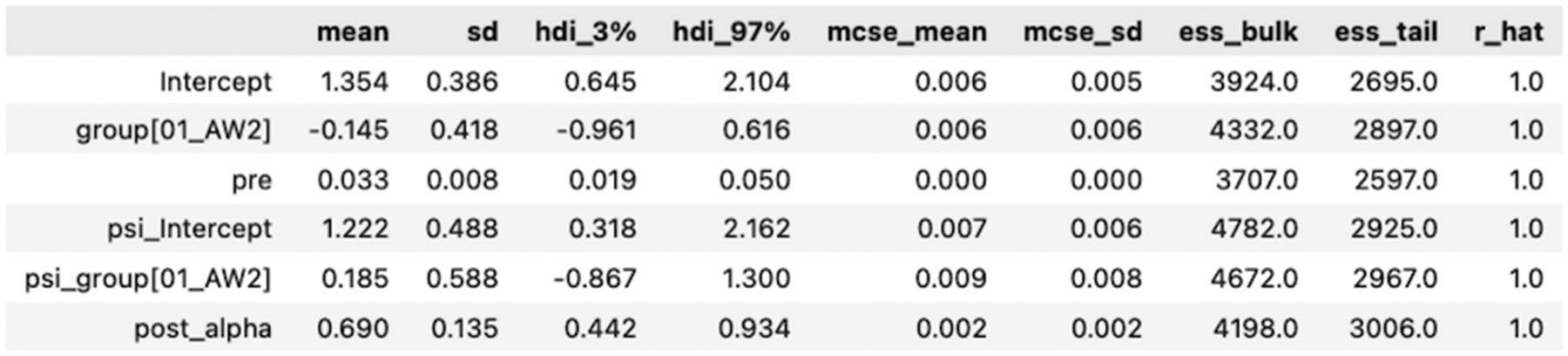
Markov Chain Monte Carlo (MCMC) summary.

The model was fit using PYMC using uninformative priors scaled to the range of the data and on the log and logit scales for the appropriate parameters. Four chains with a total of 4000 draws were sampled. The resulting Markov Chain Monte Carlo (MCMC) summary is shown in Figure 1.

The R‐hat statistic shows that our chains converged well, and sampling showed no divergences. Our Effective Samples Statistics (ESS) show that our samples have high resolution, which shows good sampling efficiency.

Bayesian ANOVA's were similarly fit to compare Starting Area (in cm^2^), Product Applications, and Treatment Days using a Gamma Likelihood, a Negative Binomial Likelihood, and a Negative Binomial Likelihood, respectively. The results are reported in Table 2.

## 3 RESULTS

The statistical analysis revealed that the group receiving RE‐AC, despite being somewhat older (71 vs. 58.6 years) had an xPAR that was on average 14.1 percentage points (95% credible interval: −1.0% to 30.12%) greater than the L‐AC group at 12 weeks. (Table 2) Further, the probability of the full wound closure in the L‐AC group was on average 0.017% percentage points greater (95% credible interval: −0.67% to 0.04%). This suggests RE‐AC and L‐AC groups are substantively similar in terms of complete wound closure, but that RE‐AC has a greater general effect on wound closure when both full and partial closure are considered.

Moreover, the average number of applications per wound in the RE‐AC group was 7.9 versus 10.6 in the L‐AC group, suggesting that RE‐AC is more than 27% more efficient in terms of general wound closure efficiency in terms of applications required. The retrospective analysis revealed a significant finding that favored RE‐AC over L‐AC in terms of treatment efficiency for wound care. Patients who were treated with RE‐AC required fewer applications of the product to achieve wound healing outcomes that were comparable with those treated with L‐AC.

## 4 DISCUSSION

The primary implication of RE‐AC's requirement for fewer applications, besides system‐wide cost savings, is improved patient comfort and convenience. Patients who need wound care treatments often experience discomfort and inconvenience associated with frequent visits for application. This is due to the discomfort of dressing changes and debridement, as well as travel required to attend the clinic visits. It is a factor in patient compliance to complete the full course of treatment. With RE‐AC, requiring fewer applications can significantly reduce this treatment burden, potentially leading to higher patient satisfaction and adherence to the prescribed treatment regimen.

This study highlights the advantage of retaining placental factors in allografts for diabetic foot wounds. The stromal components of both RE‐AC and L‐AC are conducive to fibroblast migration into the wound bed. Both processes provide collagen scaffolding and probably fibronectin stabilization. The difference is the increased available factors in membrane that is processed intact (not separated during processing) and processed with conditions that do not remove the tissue in the intermediate layers and below the chorionic basement membrane. Those factors responsible for the improved results likely include IL‐1ra, HGF, glycosaminoglycans and PDGF‐BB, among others.^37^

Fewer applications also translates into optimized resource utilization within healthcare facilities. Clinicians and healthcare providers can allocate their time and resources more efficiently, as they need to administer and monitor treatments less frequently. This can be particularly valuable in busy outpatient wound care centers where resources are at a premium.

Another notable implication is the potential for cost savings in healthcare settings. Fewer applications mean reduced consumption of wound care materials and healthcare provider time, which can result in lower treatment costs. For example, the reduction of applications required results in 27% increased efficiency and 14% less treatment days. This translates to less office visits and less product required to achieve the same result. This cost‐effectiveness aspect can be of significant interest to healthcare administrators and policymakers. The average cost to treat a DFU can be up to $50,000 per episode for advanced stage ulcers considering all costs of care.^38^ According to our results, the use of RE‐AC could reduce that cost significantly by expediting wound closure, thereby avoiding complications and reducing the frequency of necessary amniotic membrane applications.

Retrospective data retrieved from EHRs offer valuable insights for research but are prone to limitations and biases. These include missing or incomplete data, selection bias from patients seeking care or clinical practices and clinical practice guidelines, and confounding variables like demographics that can skew findings. Inconsistencies in documentation and wound measurement further challenge data reliability. Additionally, the generalizability of EHR data may be limited.

As it relates to the instrumental variables used in the study, the models were parsimonious and did not rely on numerous instrumental variables. The variables of interest consisted of surface area of the wound in square centimeters, timepoint of measurement (pre or post), and total applications over the time of study. Adjustment was made using ANCOVA in the models on observed and included covariates. However, an argument can be made that more covariates could have been included such as age, comorbidities, and prior length of treatment, which would have controlled for these variables.

The study's findings can influence clinical decision‐making by guiding healthcare professionals to consider the treatment efficiency in terms of the number of applications. RE‐AC's superiority in this regard may lead to its preference as a treatment option over L‐AC, particularly in cases where minimizing the frequency of applications is a priority.

## 5 CONCLUSIONS

This comparative analysis of (RE‐AC) and (L‐AC) in treating DFUs highlights the distinct advantages of RE‐AC, especially in terms of application efficiency and wound size reduction. This efficiency is likely due to the retention of immunomodulatory, structural and regenerative factors in the processing method of RE‐AC.^9^, ^28^

From the retrospective data analyzed from three outpatient wound care centers, RE‐AC demonstrated a marginally higher expected Percent Area Reduction (xPAR) over 12 weeks, underscoring its effectiveness in managing wound size.

A key distinction between RE‐AC and L‐AC lies in the frequency of applications required. RE‐AC's reduced application frequency not only enhances patient comfort by lessening the need for repeated treatments but also signifies a more cost‐effective and resource‐efficient approach in clinical settings. While L‐AC might offer a slight edge in achieving complete wound closure, the operational efficiency of RE‐AC makes it a preferable option in scenarios where reducing treatment frequency is crucial.

Moreover, the study reveals that RE‐AC's balance between efficacious treatment and operational practicality makes it a compelling choice for DFU management. Its ability to effectively reduce wound size with fewer interventions presents a significant advantage in both patient experience and healthcare resource management.

Further investigation of the long‐term effects of RE‐AC and L‐AC treatment on wound healing and patient outcomes is ongoing. The data from long‐term studies will be used to determine the cost‐effectiveness of these treatments in different healthcare settings. Comparative analysis of RE‐AC versus SOC is ongoing in both retrospective study and RCT. The retrospective data is due to be submitted in 2024. Lack of access to L‐AC product hinders the ability to include it in clinical trial data, but future studies will be possible as larger long‐term data is generated. Gaining this information will augment reporting for cost savings over long‐term care.

In conclusion, while both RE‐AC and L‐AC are effective in the treatment of DFUs, RE‐AC's efficiency in application and comparable efficacy in wound size reduction positions it as a more advantageous option in many clinical cases. This study underscores the importance of evaluating both clinical outcomes and practical aspects of treatment in selecting the most suitable intervention for DFUs.

## AUTHOR CONTRIBUTIONS

**Robert G Frykberg**: Methodology; writing—original draft; writing—review & editing. **Zwelithini Tunyiswa**: Formal analysis; writing—original draft; writing—review & editing.

## CONFLICT OF INTEREST STATEMENT

RGF and ZT received no direct funding other than support in the preparation of this manuscript. Neither RGF nor ZT have any formal relationship, past or present, with BioStem Technologies. Both authors have read and approved the final version of the manuscript. RGF had full access to all of the data in this study and takes complete responsibility for the integrity of the data and the accuracy of the data analysis.

## TRANSPARENCY STATEMENT

The lead author Robert G. Frykberg affirms that this manuscript is an honest, accurate, and transparent account of the study being reported; that no important aspects of the study have been omitted; and that any discrepancies from the study as planned (and, if relevant, registered) have been explained.

## Data Availability

Data and methods are available for review upon written request to the contributing author (RGF). The data that support the findings of this study are available on request from the corresponding author. The data are not publicly available due to privacy or ethical restrictions.
